# Aloes in Hemorrhoids

**Published:** 1873-05

**Authors:** 


### ALOES IN HEMORRHOIDS.

Prof. Fordyce Barker, in the *American Practitioner*, on the treatment of hemorrhoids in pregnancy, says: "When hemorrhoids are developed during the latter period of pregnancy, the indications are obviously to counteract the constipation or the diarrhœa, and to stimulate and to restore the tonicity of the hemorrhoidal veins. The inquiry will then naturally suggest itself, have we any agent or combination of agents in the *materia medica* capable of effecting these results? I know of no article which so clearly and positively produces these two results as aloes, and on this I have mainly relied. I am well aware that the general voice of the profession is against the use of aloes when there is any tendency to hemorrhoids."

We are enabled to add our testimony to that of Prof. Barker's in the use of aloes in the condition referred to. We would, also, in this connection, call attention to the use of aloes in hemorrhoids, from whatever cause, occurring in the male or female. Given in small does we get its mild, stimulant action upon the dilated and distended vessels, and hence a restoration of tonicity. We give the remedy in one grain doses, two or three times a day, combined usually with a grain of the sulphate of iron and one-fourth of a grain of extract belladonna. This combination will rarely disappoint us in relieving both the attendant constipation and the hemorrhoids.

